# Inverter control for wind–solar–storage based on distributed generation and virtual synchronous machines

**DOI:** 10.1371/journal.pone.0359162

**Published:** 2026-10-27

**Authors:** Heng Cao

**Affiliations:** Ningxia Branch, PowerChina Renewable Energy Co., Ltd., Yinchuan, China; Balikesir Universitesi, TÜRKIYE

## Abstract

To enhance the operational stability of the power system and address the issues of weak inertia support and sluggish dynamic response of traditional inverter control strategies when dealing with complex scenarios involving a high proportion of new energy sources, a new intelligent control strategy has been proposed. This study adopts the VSG control strategy as the core technology for the regulation of wind-solar-storage power generation inverters, and divides the dynamic response process into acceleration period, deceleration period, reverse acceleration period and reverse deceleration period. Different control strategies are adopted for different system states at different stages. Additionally, the DDPG algorithm is introduced, and the single DDPG agent is extended to a multi-agent interactive deep deterministic policy gradient algorithm. Finally, the multi-agent collaborative optimization is achieved in a “centralized training and decentralized execution” form. The research results show that in the case of sudden load increase, the voltage deviation at the grid connection point is stabilized at 371.7 V, and the voltage recovery time is significantly shorter than the comparison strategy. At the same time, the time-weighted absolute error integral reaches optimal values of 1.82 V/s^2^ and 1.85 V/s^2^ in scenarios of sudden load increase and sudden drops in photovoltaic power, and the strategy efficiency reaches 97.83%, and the steady-state accuracy reaches 99.63%, demonstrating superior control capabilities. In the three typical fault conditions of practical application, the average frequency deviation of the control strategy was 0.197 Hz, the number of protection lockouts was 0.3 times, the average depth of voltage sag was 24.63%, the average recovery time was 1.21 hours, and the number of abnormal warnings did not exceed 5 times. The proposed framework for coordinated wind, solar, and storage regulation, based on distributed power generation and virtual synchronous machine, can effectively support the safe, flexible, and efficient operation of the new power system under high penetration of renewable energy.

## 1. Overview

With the implementation of the “carbon peak, carbon neutrality” strategy, renewable distributed power generation technologies such as wind energy and solar energy have grown rapidly and have become an important part of future energy sources [1,2]. However, the randomness and volatility of wind power and photovoltaic power pose challenges to the safety and stability of the power grid. Traditional power systems rely on the rotational inertia and damping of synchronous generators to suppress disturbances and maintain stability [3]. However, distributed power sources such as wind and solar energy are connected to the grid through inverters, lacking inertia, which reduces the equivalent rotational inertia of the power grid and weakens its anti-interference ability. Therefore, maintaining the stability of distributed power sources in the power grid is a core issue that needs to be urgently addressed [4,5]. Elangovan P et al. proposed a control strategy combining fuzzy logic and adaptive generalized integrator to address the power quality issues in photovoltaic grid-connected systems. This scheme dynamically adjusts parameters through fuzzy logic to effectively adapt to grid fluctuations. Experimental results show that this method can significantly improve the current quality of the power grid [6]. Compared with the method in reference [6], this study has obvious shortcomings in parameter adaptability and multi-condition robustness: the fuzzy rules are determined based on manual experience, which is difficult to cover all scenarios of disturbances; the unmodeled dynamic evolution process of grid inertia leads to a delayed response during voltage recovery at the initial stage; and the introduction of a collaborative optimization mechanism is absent, resulting in control conflicts under multi-source coupling disturbances. In contrast, the proposed multi-agent Deep Deterministic Policy Gradient-Virtual Synchronous Generator (DDPG-VSG) framework in this paper achieves millisecond-level adaptive reconstruction of control parameters through online learning of the system inertia-damping coupling relationship. The research team of Liu H addressed the voltage over-limit and network loss problems caused by photovoltaic grid connection and proposed a multi-objective particle swarm algorithm that integrates adaptive networks and roulette wheel mechanism. Simulation experiments show that this algorithm significantly improves the voltage fluctuation characteristics while ensuring the distribution of the solution set [7]. The method in reference [7] has advantages in multi-objective collaborative optimization, but its particle swarm algorithm has a slow convergence speed and does not model the dynamic response process of system inertia, resulting in a longer voltage recovery time during transient disturbances such as load sudden changes; moreover, the randomness introduced by the roulette wheel mechanism weakens the control certainty and is difficult to meet the strict requirements of the new power system for millisecond-level response accuracy. In contrast, the multi-agent DDPG-VSG framework proposed in this paper builds a global inertia-damping coupling knowledge graph through centralized training and ensures decentralized execution to guarantee the independence and real-time response of each unit in dual disturbance conditions such as sudden drop in photovoltaic output and sudden increase in load, maintaining high dynamic response accuracy and voltage recovery speed. Junior L R et al. designed a 3.5kW single-phase voltage source inverter for photovoltaic grid connection using an Inductor-Capacitor-Inductor (LCL) filter structure. Through experiments, it was found that this transformer can effectively regulate grid voltage fluctuations and provide reactive power support [8]. Although existing studies can effectively improve power quality and voltage stability, they fail to consider the core challenge of the equivalent rotational inertia of the system decreasing due to the high proportion of new energy grid connection.

Existing research improves power quality and voltage stability but fails to address declining system equivalent rotational inertia from high renewable energy. VSG control methods simulate synchronous generators to provide inertia and damping support. Ghazzawi N A et al. proposed a VSG strategy for photovoltaic inverters, managing power to respond to grid fluctuations; simulations show voltage control [9]. However, reference [9] uses offline-tuned parameters, lacking online perception and feedback, causing overshoot or insufficient damping during fluctuations, hindering stability-accuracy balance. This paper's multi-agent DDPG-VSG framework achieves millisecond-level parameter optimization and dynamic response via real-time ROCOF and voltage deviation perception, generating coordinated inertia-damping instructions. Utkarsha P et al. proposed a seamless switching VSG strategy for frequency regulation without extra storage, using load reduction MPPT; experiments show stable operation and frequency disturbance solution [10]. But reference [10] does not model inertia dynamics, leading to delayed response and insufficient support under multi-source disturbances. In contrast, this study achieves millisecond-level coordinated inertia-damping response under multi-source disturbances by online learning inertia-damping coupling.

Existing methods have defects like fixed parameters and insufficient adaptability, making it hard to balance inertia support and dynamic response accuracy under high renewable energy penetration. Fixed-parameter VSG is prone to frequency regulation lag or overshoot during disturbances like load changes or renewable output drops. The research designs an adaptive adjustment rule for VSG inertia and damping coefficients based on dynamic response, introducing an improved Deep Deterministic Policy Gradient (DDPG) algorithm. This combines VSG control with multi-agent DDPG in a centralized training-decentralized execution architecture. Innovations: (1) Theoretical: A VSG multi-agent reinforcement learning framework for high-renewable grids, modeling VSG electromechanical transients and DDPG policy updates under Lyapunov stability constraints, proving convergence and robustness; (2) Technological: A physical model-guided policy network initialization method, embedding VSG differential equations into DDPG Actor network weights for action priors; (3) Application: A lightweight edge deployment architecture supporting real-time inference on ARM+FPGA chips. DDPG is chosen for efficient nonlinear strategy learning in continuous action spaces, suitable for real-time inertia-damping coordination; its deterministic output avoids random exploration disturbances, ensuring grid safety; distributed multi-agent architecture enables online optimization without global communication. Contributions: (1) The research deeply couples the multi-agent DDPG algorithm with the VSG physical model to achieve online joint optimization of inertia and damping parameters. Compared with existing studies that only optimize a single parameter and ignore the multi-machine synergy effect, this approach achieves global optimal parameter configuration under multi-machine collaboration, thereby improving the frequency response performance of the system under complex disturbances; (2) Experience replay sharing reduces communication overhead while ensuring convergence; (3) Adaptive VSG inertia-damping adjustment solves frequency issues during disturbances.

## 2. Materials and methods

### 2.1. Adaptive rule design for VSG control strategy parameters

Distributed generation units like wind, solar, and storage connect via traditional inverters but lack flexible regulation, failing to respond to grid dynamics and causing stability issues [11]. VSG technology emulates the characteristics of synchronous generators to enhance system stability. Thus, this paper adopts VSG control as the core for inverter control in wind-solar-storage systems, enhancing grid support by emulating synchronous generator operation. VSG control uses algorithms to mimic key external characteristics of traditional synchronous generators, as shown in Fig 1.

**Fig 1:**
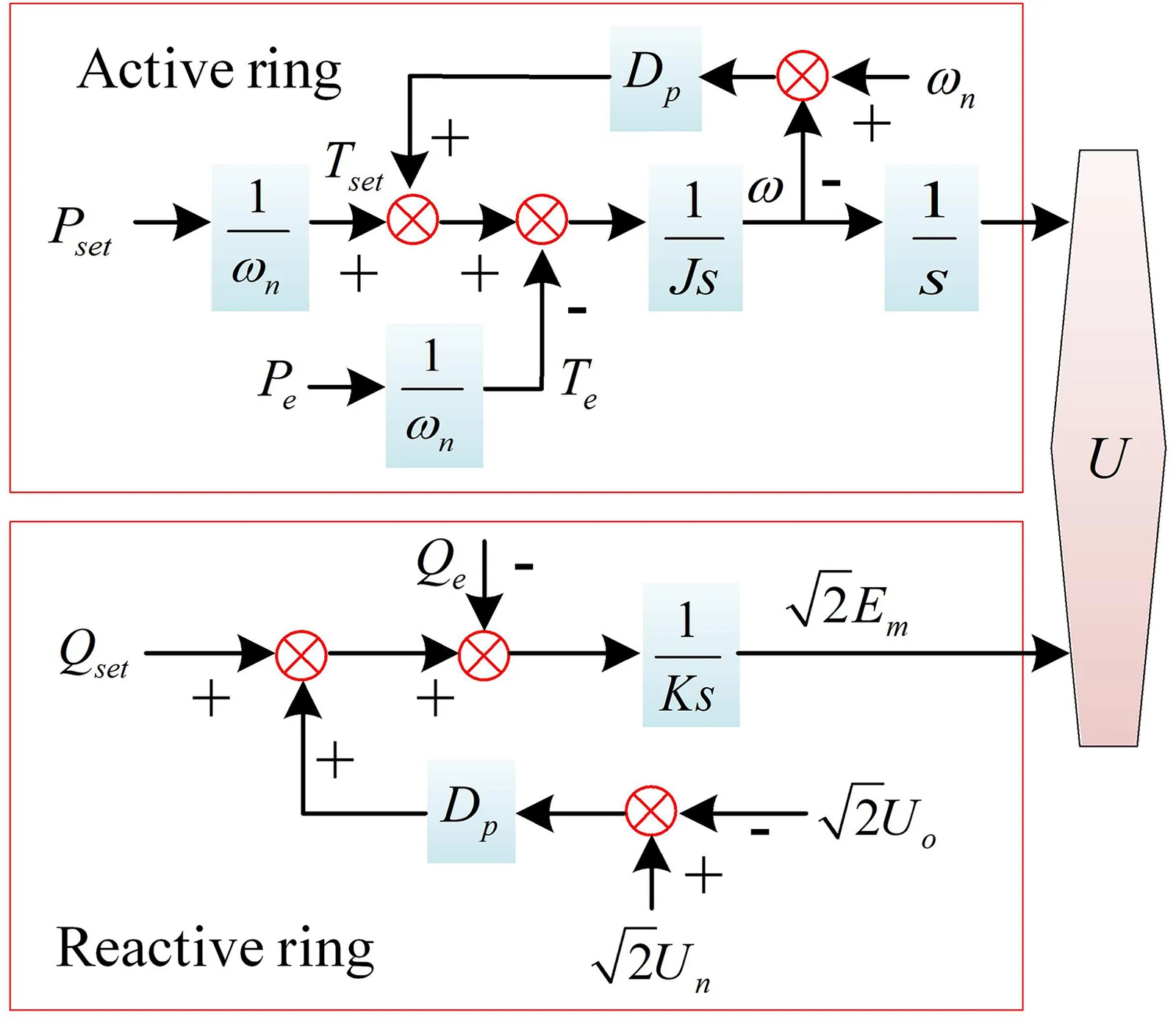
VSG control principle diagram.

Fig 1 shows that VSG mainly includes two core parts: active-frequency control and reactive-voltage control. Active-frequency control introduces a rotor motion equation of the synchronous generator. It calculates the power deviation, generates the voltage phase, and provides virtual inertia and damping for the inverter. This enables the inverter to autonomously adjust its active power output according to grid frequency and suppress frequency fluctuations. Reactive-voltage control emulates the excitation regulation characteristic of the synchronous generator. It uses the droop control principle to adjust reactive power deviation and then changes the output voltage amplitude to control grid voltage [12,13]. Active-frequency control is the key to VSG technology. The expression of the rotor motion equation is shown in Equation (1).


$$\begin{array}{c} J\frac{d\left(\omega -\omega_{\text{0}}\right)}{dt}=\frac{P_{m}}{\omega_{\text{0}}}-\frac{P}{\omega_{\text{0}}}-T_{D}=\frac{P_{m}}{\omega_{\text{0}}}-\frac{P}{\omega_{\text{0}}}-D\left(\omega -\omega_{\text{0}}\right) \end{array}$$ (1)

In Equation (1), ω represents the virtual angular speed of the VSG. $\omega_{\text{0}}$ represents the rated angular speed. $P_{m}$ represents the virtual mechanical power. P represents the electromagnetic power. D represents the virtual damping coefficient. $T_{D}$ represents the prime mover torque of the actual synchronous generator. When the system load increases suddenly and the voltage frequency decreases, $P_{m}$ becomes larger immediately and the virtual rotor begins to slow down. During this process, the virtual kinetic energy converts into electrical energy and sends it to the grid, preventing the frequency from dropping quickly. When the system load decreases, the opposite situation occurs. The VSG absorbs excess power, speeds up its virtual rotor, and prevents the frequency from rising rapidly [14]. The reactive-voltage control uses the droop control theory. Its expression is shown in Equation (2).


$$\begin{array}{c} U_{ref}=U_{N}+K_{q}\left(Q_{ref}-Q\right) \end{array}$$ (2)

In Equation (2), $U_{N}$ represents the rated voltage. $Q_{ref}$ represents the given reactive power. Q represents the actual reactive power output. $K_{q}$ represents the reactive-voltage droop coefficient. When the grid voltage drops, the VSG outputs more reactive power Q to support the voltage. This increases its output voltage and injects reactive power into the grid. When the voltage increases, the VSG reduces reactive power output [15,16]. VSG synthesizes the frequency, phase and amplitude information from the two control loops through vector synthesis. The synthesis equation is shown in Equation (3).


$$\begin{array}{c} \left\{ \begin{array}{c} U_{oref}=U_{ref}cos\left(\phi \right)\\ U_{oref}=U_{ref}cos\left(\phi -120^{\circ }\right)\\ U_{oref}=U_{ref}cos\left(\phi +120^{\circ }\right) \end{array}\right. \end{array}$$ (3)

In Equation (3), $U_{ref}$ represents the reference output voltage of the VSG. The synthesized three-phase voltage command is obtained and used to achieve accurate inverter control. When the VSG operates in the grid-connected mode, the configuration and fixed setting of virtual parameters become key factors that limit its control performance. If the parameters are too high, the frequency adjustment becomes slow. If they are too low, oscillation occurs easily and frequency support becomes weak. In high-renewable power systems, fixed parameters cannot adapt to changes, and the system cannot match grid fluctuations and transient faults, leading to regulation mismatch [17,18]. To solve the problem of non-adaptive parameters, this paper puts forward an adaptive inverter control strategy for wind–solar–storage based on the dynamic response characteristics of the VSG. It realizes the real-time adjustment of virtual inertia and damping. The dynamic response characteristic curve of the VSG is shown in Fig 2.

**Fig 2:**
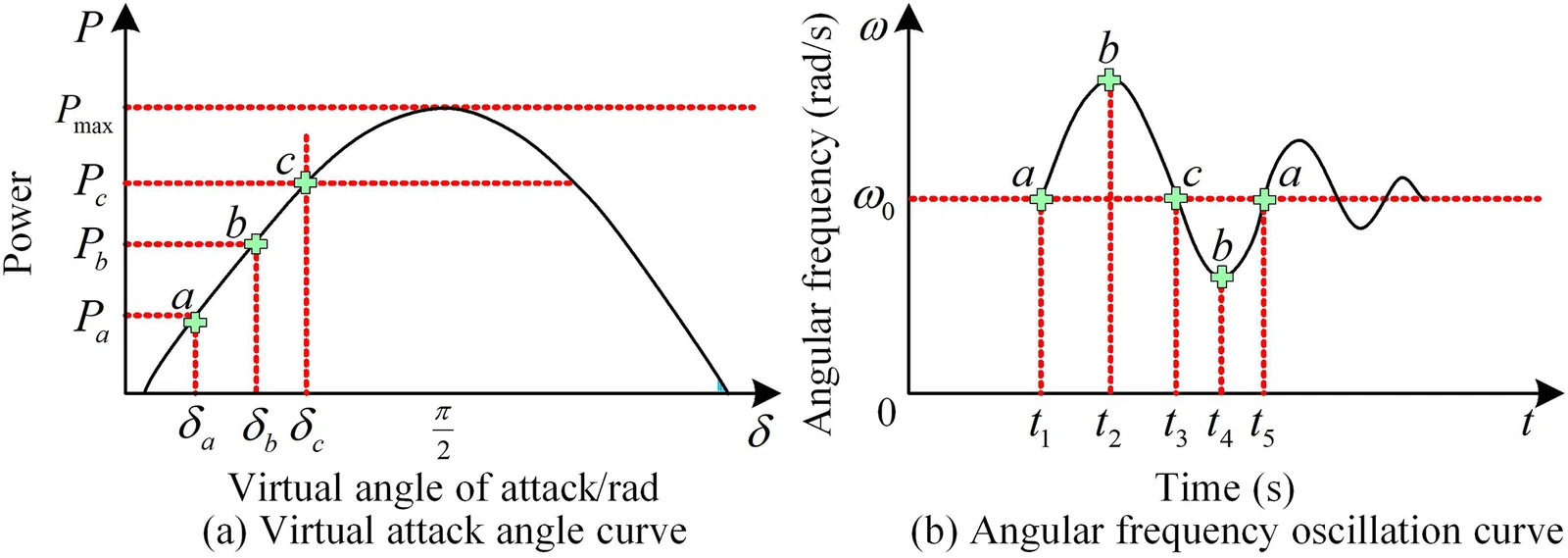
VSG dynamic response characteristic curve.

Points a, b, and c in Fig 2 correspond to points on virtual attack angle curves, with a for steady-state operation and b for transient overload critical point. Fig 2(a) shows dynamic coupling between VSG active power and power angle. Under power disturbances, frequency recovery exhibits second-order system characteristics. For example, when reference power jumps from a to Pb, the trajectory from a to c shows decaying oscillation and convergence, as reflected in Fig 2(b). Based on these dynamics, the response process is divided into deceleration, reverse acceleration, and reverse deceleration periods. System state varies by stage, requiring differentiated control: in acceleration, inertia increases and damping rises; in deceleration, inertia decreases and damping remains high; in reverse acceleration, large inertia is configured and damping moderately reduced; in reverse deceleration, small inertia and low damping are adopted to enhance dynamic response and stability. Based on these rules, an adaptive control strategy for inertia and damping is built, with flow shown in Fig 3.

**Fig 3:**
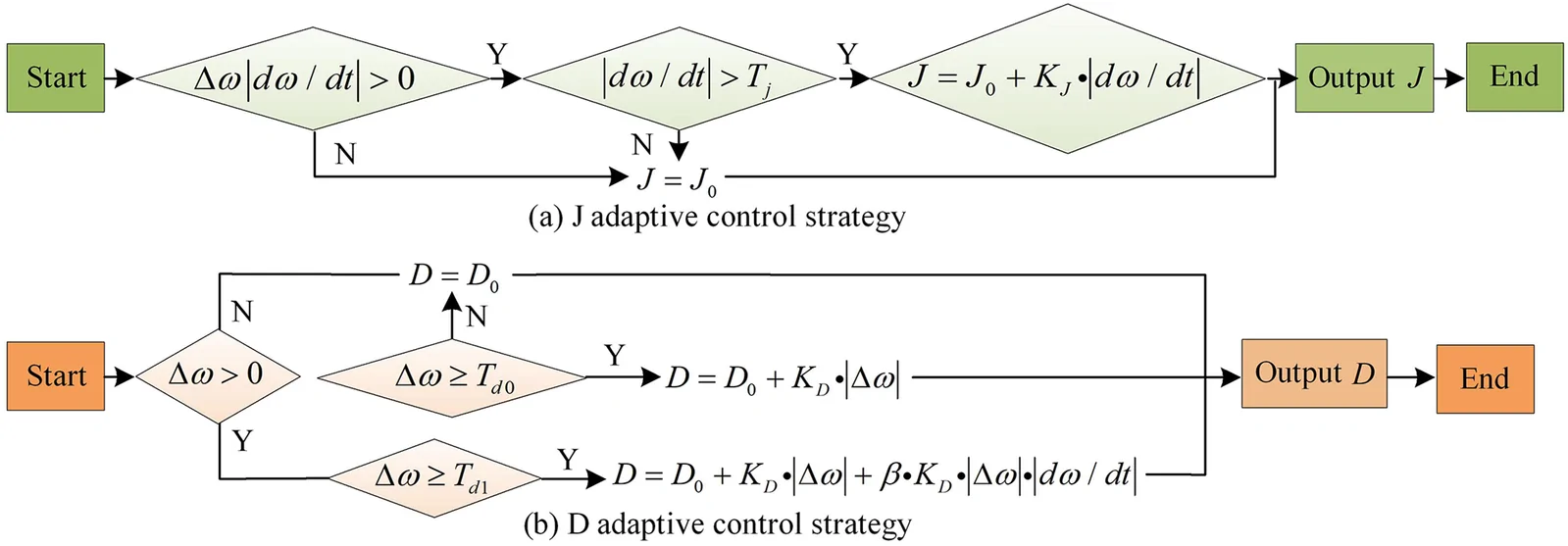
Adaptive parameter control strategy flowchart.

As illustrated in Fig 3, the adaptive inertia control increases inertia when the product of the angular-frequency deviation and its rate of change is positive. It decreases inertia when the product is negative. The mathematical expression is shown in Equation (4).


$$\begin{array}{c} \left\{ \begin{array}{c} J=J_{\text{0}}\;\;\;\;\;\;\;\}\Delta \omega \left(d\omega /dt\right)<0\cup d\omega /dt\leq T_{j}\\ J=J_{\text{0}}+K_{J}•\left|d\omega /dt\right|\;\}\Delta \omega \left(d\omega /dt\right)>0⋂d\omega /dt>T_{j} \end{array}\right. \end{array}$$ (4)

Fig 3(b) shows that the adaptive damping control increases damping when the angular-frequency difference is greater than 0 and decreases damping when it is less than 0. The mathematical expression is shown in Equation (5).


$$\begin{array}{c} \left\{ \begin{array}{c} D=D_{\text{0}}\;\;\;\;\;\;\;\;\;\;\;\;\;\;\}\Delta \omega <0\cup \left|\Delta \omega \right|\leq T_{d0}\\ D=D_{\text{0}}+K_{J}•\left|\Delta \omega \right|\;\;\;\;\;\;\;\}\Delta \omega >0\cap T_{d0}<\left|\Delta \omega \right|\leq T_{d1}\}\\ D=D_{\text{0}}+K_{J}•\left|\Delta \omega \right|+\beta •K_{D}•\left|\Delta \omega \right|•\left|d\omega /dt\right|\}\Delta \omega >0\cap \left|\Delta \omega \right|>T_{d1}\} \end{array}\right. \end{array}$$ (5)

Equation (5) shows that when the angular-frequency difference is in the $\left(0,T_{d0}\right)$ range, the deviation is small and the system uses small damping for faster response. When the angular-frequency difference is in the $\left(T_{d0},T_{d1}\right)$ range, the system is in the middle of a disturbance and should use higher damping. When the angular-frequency difference is greater than $T_{d1}$, the frequency deviation is large and requires large damping to suppress the deviation. Based on these rules, this paper sets adaptive adjustment rules for VSG inertia and damping to solve the problem caused by fixed parameters.

### 2.2. Construction of wind–solar–storage inverter control strategy based on adaptive parameter control

The adaptive parameter control strategy in this paper realizes dynamic parameter adjustment. However, distributed power systems differ from traditional centralized systems. They need to be deployed at the user side or close to the user. They have dispersed distribution, small scale and variable power flow. These characteristics make fixed rules difficult to achieve global optimal control. DDPG is a reinforcement learning algorithm that solves continuous action control problems. It combines the features of a deep Q network and the policy gradient method. With the “actor–critic” framework, the agent learns how to output continuous control actions in complex and dynamic environments [19,20]. Therefore, this paper introduces the DDPG algorithm and transforms rule-based control into a data-driven intelligent control framework. A DDPG algorithm was designed targeting the core objectives of power system frequency control and the dynamic characteristics of VSGs. The state space is defined as $o_{t}=\left[△f_{t},\frac{d\left(△f\right)}{dt},P_{t}\right]$. The frequency deviation △f and its rate of change directly correspond to the angular velocity and angular acceleration in the VSG rotor motion equation, serving as the core physical quantities determining inertia and damping response. The output power $P_{t}$ characterizes the system's power balance state, enabling the agent to perceive disturbance magnitude. The action space directly outputs adjustment commands $a_{t}=\left[△J_{t},△D_{t}\right]$ for virtual inertia and damping coefficients. The reward function is designed to $r_{t}=-\left(\alpha |△f_{t}|+\beta \int \left|△f_{t}\right|dt\right)$, aims to synergistically optimize steady-state accuracy and dynamic speed in frequency control. The first term penalizes instantaneous frequency deviations, directly addressing the primary requirement for power system frequency stability; the second term penalizes cumulative deviations, incentivizing the controller to accelerate transient recovery processes. The weighting coefficients α and β are determined through parameter sensitivity analysis to balance response speed and overshoot. The control flow of the inverter control strategy for VSG wind–solar–storage based on DDPG is shown in Fig 4.

**Fig 4:**
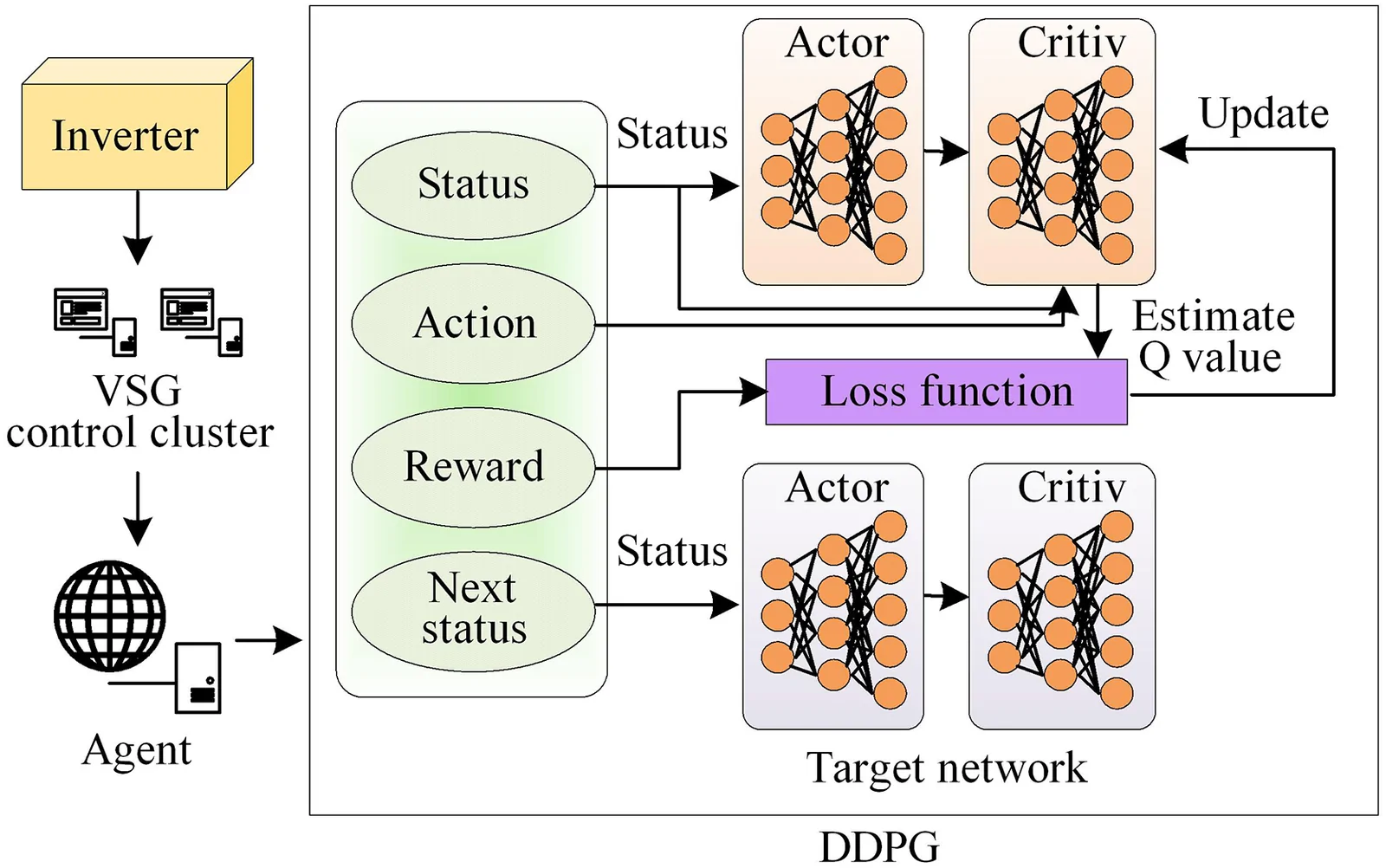
Control strategy flow based on the DDPG algorithm.

Fig 4 shows that the VSG controller works as an agent. Each agent adjusts the inertia and damping according to the DDPG algorithm. In the DDPG algorithm, the Actor network produces continuous control actions based on the state of the environment. The Critic network evaluates the action value and optimizes the policy iteratively [21,22]. Many advanced control strategies have been proposed in existing research, including TD3. The motivation for choosing the DDPG algorithm in this study is that it has a more stable policy update mechanism and higher sample utilization efficiency in continuous control tasks, especially suitable for real-time control scenarios with frequent power fluctuations and strict response time requirements in wind–solar–storage systems. Meanwhile, DDPG has stronger modeling capabilities for high-dimensional state spaces. Although TD3 can solve the shortcomings of DDPG, its dual Critic structure and delayed update mechanism significantly increase online inference latency, which can easily cause control lag under millisecond level response constraints in wind solar storage systems. In contrast, DDPG has lower computational overhead and stronger real-time adaptability while ensuring convergence, which aligns more closely with the dual requirements of dynamic robustness and engineering deployability in this study. The current Actor network updates its policy gradient by following Equation (6) [23].


$$\begin{array}{c} \nabla J\left(\theta \right)\approx \frac{\text{1}}{N}\sum_{i}\nabla aQ\left(s,a|\theta^{Q}\right)|{\;}_{s=s_{i},a=\mu \left(s_{i}\right)}\nabla_{\theta^{\mu }}\mu \left(s|\theta^{\mu }\right)|{\;}_{s_{i}} \end{array}$$ (6)

In Equation (6), N represents the sample size. s represents the agent state. $s_{i}$ represents the current state. a represents the current action of the Actor network. $\theta^{Q}$ indicates the parameters of the Critic network. $\theta^{\mu }$ indicates the parameters of the Actor network. μ indicates the action output of the Actor network. Q represents the function of the agent executing an action from a given state. The update of the Critic network loss function follows Equation (7) [24].


$$\begin{array}{c} L\left(\theta^{Q}\right)=\frac{\text{1}}{N}{\sum_{i}\left(y_{i}-Q\left(s,a|\theta^{Q}\right)\right)}^{\text{2}} \end{array}$$ (7)

In Equation (7), $y_{i}$ represents the target value, which is shown in Equation (8).


$$\begin{array}{c} y_{i}=r_{i}+\gamma Q^{\prime }\left(s_{i+1},\mu (s_{i+1}|\theta^{\mu^{\prime }})|\theta^{Q^{\prime }}\right) \end{array}$$ (8)

In Equation (8), $r_{i}$ represents the immediate reward. γ represents the discount factor. $s_{i+1}$ represents the next state of the agent. The update rule of the Critic network parameter $\theta^{Q}$ follows Equation (9) [25].


$$\begin{array}{c} \theta^{Q}\leftarrow \theta^{Q}+nc\nabla_{\theta^{Q}}L\left(\theta^{Q}\right) \end{array}$$ (9)

In Equation (9), nc represents the learning rate of the Critic network. The update rule of the Actor network parameter $\theta^{\mu }$ follows Equation (10).


$$\begin{array}{c} \theta^{\mu }\leftarrow \theta^{\mu }+na\nabla_{\theta^{\mu }}L\left(\theta^{\mu }\right) \end{array}$$ (10)

In Equation (10), na represents the learning rate of the Actor network. $\theta^{Q}$ and $\theta^{\mu }$ update based on a soft update mechanism. This mechanism introduces an update coefficient between 0 and 1 to control the update speed of the target network. The update rule is shown in Equation (11).


$$\begin{array}{c} \left\{ \begin{array}{c} \theta^{Q^{\prime }}\leftarrow \tau \theta^{Q}+\left(1+\tau \right)\theta^{Q^{\prime }}\\ \theta^{\mu^{\prime }}\leftarrow \tau \theta^{\mu }+\left(1+\tau \right)\theta^{\mu^{\prime }} \end{array}\right. \end{array}$$ (11)

In Equation (11), τ represents the update coefficient. Although DDPG can effectively handle the continuous control problem of VSG inertia and damping parameters, when multiple VSG units operate in parallel within the system, each DDPG agent can only make independent decisions based on its local environmental state. It cannot perceive the control actions of other parallel units. This results in a lack of connectivity between VSG units, preventing the achievement of system-level cooperative optimization. This “information silo” state causes conflicting or suboptimal adaptive adjustment strategies among VSGs, preventing system-level cooperative optimal frequency control. To address this, this study proposes a Multi-Agent Interactive Deep Deterministic Policy Gradient (MAIDDPG) algorithm. Its core innovation lies in introducing a collaborative architecture featuring “centralized training and decentralized execution,” aiming to resolve the challenge of cooperative decision-making in distributed multi-VSG systems. The structure of the MAIDDPG is shown in Fig 5.

**Fig 5:**
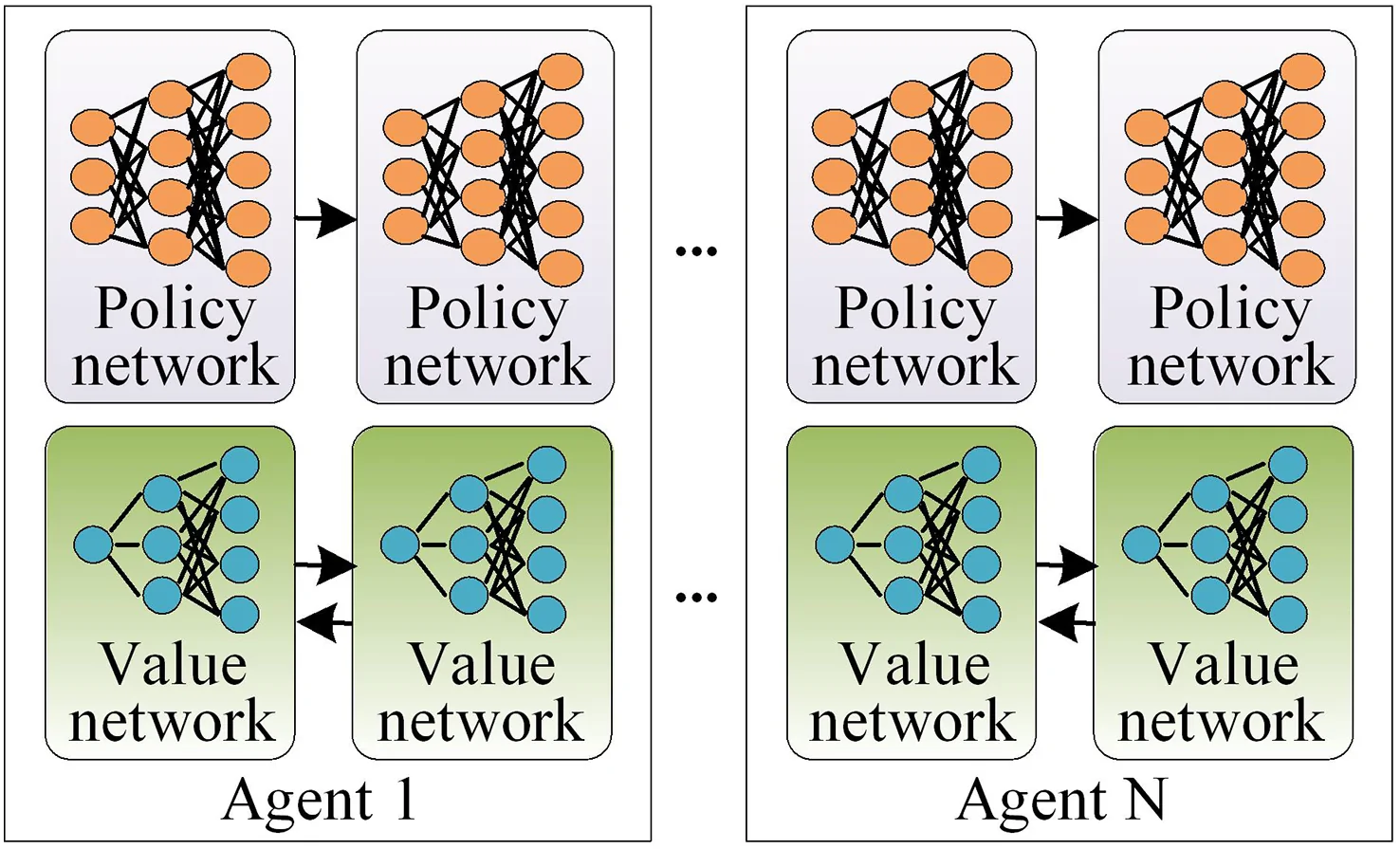
Structure of the MAIDDPG algorithm.

Fig 5 shows that MAIDDPG adopts centralized training and decentralized execution. During training, the Critic network of each VSG agent can access the state and action information of all other VSGs in the system. This enables agents to learn to make decisions beneficial to the overall system while fully understanding the impact of other agents’ behaviors, thereby acquiring cooperative strategies. In the MAIDDPG algorithm, the Actor network adopts a three-layer fully connected structure, with the input being the local observation state and the output being the continuous control action; the Critic network takes the concatenation of the global state and the actions of each agent as the input and outputs the corresponding Q value. All networks use the ReLU activation function and introduce Batch Normalization to accelerate convergence. The selection of the number of hidden layers and the number of nodes needs to strike a balance between the model's expressive power and the risk of overfitting. The three-layer structure combines the nonlinear fitting ability and training stability. The number of nodes is set based on the experience of the state-action space dimension to be 128–256. Insufficient nodes lead to underfitting, while too many nodes cause slow convergence and policy oscillation. The research obtained the optimal configuration through grid search and ablation experiments: the number of hidden layers is 3, with 256 nodes in each layer. During execution, each VSG agent can independently make optimal decisions based solely on local observations, without requiring real-time, high-speed communication with other units. This mechanism enables the adaptive adjustment strategies for the inertia and damping parameters of multiple VSGs to coordinate with each other, suppressing system frequency fluctuations from a global perspective to achieve collective optimization. Assume there are N VSG agents in the entire power grid system. Each agent i has an Actor network and a Critic network. The Critic network updates its parameters by minimizing the temporal-difference error, as shown in Equation (12).


$$\begin{array}{c} \Gamma \left(\theta_{i}^{Q}\right)=E_{\left(x,a,r,x^{\prime }\right)}\left[Q_{i}^{\mu }{\left(x,a_{\text{1}},...a_{N}-y_{i}\right)}^{\text{2}}\right] \end{array}$$ (12)

In Equation (12), x represents the global state. $x^{\prime }$ represents the next global state. The Actor network updates its parameters along the direction that increases the Q value. It maximizes the expected return, as shown in Equation (13).


$$\begin{array}{c} \nabla \theta_{i}^{\mu }J\left(\mu_{i}\right)\approx E_{\left(x,a\right)}\left[\nabla \theta_{i}^{\mu }\mu_{i}\left(a_{i}|o_{i}\right)\nabla a_{i}Q_{i}^{\mu }\left(x,a_{\text{1}},...a_{N}\right)\left|{\;}_{a_{i}=\mu_{i}\left(o_{i}\right)}\right|\right] \end{array}$$ (13)

In Equation (13), $\nabla \theta_{i}^{\mu }\mu_{i}\left(a_{i}|o_{i}\right)$ represents the gradient of the action with respect to the parameters of the Actor network. $\nabla a_{i}Q_{i}^{\mu }\left(\right)$ represents the gradient of the Q value with respect to the current action of the agent. The adaptive parameter control flow for VSG based on the MAIDDPG algorithm is shown in Fig 6.

**Fig 6:**
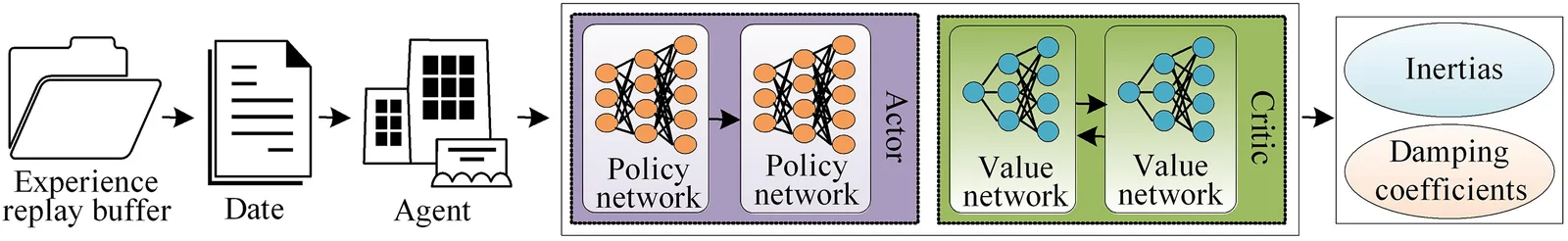
Adaptive parameter adjustment flow based on the MAIDDPG algorithm.

Fig 6 shows that the MAIDDPG-based adaptive parameter control strategy treats each VSG unit as an agent. During operation, each VSG collects key state variables such as frequency deviation, frequency rate of change and output power through local sensors. These variables are sent into the Actor network, which generates real-time adjustment commands for the virtual inertia and damping. To keep the virtual inertia and damping within a reasonable range, this paper uses a 30 kVA rated capacity and sets the damping coefficient range to [12.16, 30.4]. The damping ratio is set to the interval [0.7, 1] to balance overshoot and setting time. The mathematical expression is shown in Equation (14).


$$\begin{array}{c} \frac{D^{\text{2}}\omega_{\text{0}}}{{\text{2}}^{\text{2}}S_{E}}\leq J\leq \frac{D^{\text{2}}\omega_{\text{0}}}{1.{\text{4}}^{\text{2}}S_{E}} \end{array}$$ (14)

The reward function includes frequency deviation reward and steady-state time reward. Its expression is shown in Equation (15).


$$\begin{array}{c} R=r_{f}+r{\;}_{ts}\; \end{array}$$ (15)

In Equation (15), $r_{f}$ represents the frequency deviation. $r{\;}_{ts}\;$ represents the steady-state time. The reward function proposed in the study draws on the quantitative approach to dynamic response quality in classical control theory, but innovatively couples frequency deviation with the time required for the system to reach steady state, avoiding the imbalance of adjustment tendency caused by a single indicator; Unlike most studies that only use deviation integral (IAE/ISE) as a reward, this design explicitly introduces a steady-state time constraint term to enhance the agent's decision preference for rapid convergence of transient processes [26]. In summary, this paper first designs a rule-based adjustment mechanism for VSG inertia and damping to solve the limitation of fixed parameters. It then introduces the DDPG algorithm and expands it to the multi-agent interactive MAIDDPG algorithm to realize system-level coordinated optimization. Finally, it combines the MAIDDPG algorithm with the VSG to form the inverter control strategy for wind–solar–storage based on the Virtual Synchronous Generator-Multi-Agent Interactive Deep Deterministic Policy Gradient (VSG-MAIDDPG). This strategy improves the dynamic response of the VSG-based inverter control [27,28]. The flow of the VSG-MAIDDPG control strategy is shown in Fig 7.

**Fig 7:**
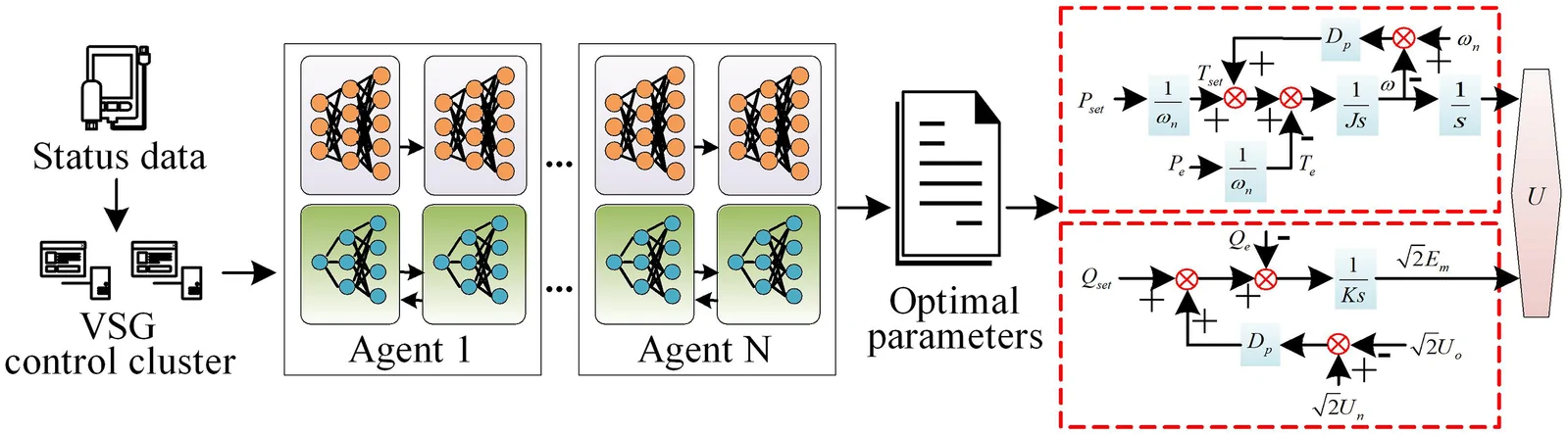
Flow diagram of the VSG-MAIDDPG control strategy.

As shown in Fig 7, The VSG-MAIDDPG strategy collects grid frequency and power as state parameters, optimized by MAIDDPG to adjust VSGs’ virtual inertia and damping, with results transmitted to controllers for individualized configuration. Each VSG calculates reference power via active frequency control and tracks voltage and current to support grid frequency. Small-signal analysis with a linearized model verifies stability; MAIDDPG dynamically adjusts parameters to change system dynamics. For a dual-VSG system under sudden load increase, damping ratio increases from 0.174 to 0.264 (52% rise), moving the main pole to enhance damping and transient decay, ensuring small-signal stability. For scalability, the strategy is deployed on distributed edge nodes using model pruning and knowledge distillation to reduce communication overhead and computational delay, with federated learning enabling multi-region collaborative updates. On an HIL platform, OPAL-RT and dSPACE simulate disturbance scenarios with real devices. Experiments show adaptive adjustment within 150ms, maximum frequency deviation reduced by 38.6%, recovery time shortened to 420ms.

The MAIDDPG agent completed the training process in a total of 3.2 hours, converging at the 847th round. The final reward value stabilized at −1.03 ± 0.07. The rated power of the wind power, photovoltaic and energy storage units was 2.5 MW, 1.8 MW and 1.2 MW/2.4 MWh respectively. The initial value of the VSG virtual inertia constant was set at 8s, and the damping coefficient was 15. The communication delay constraint was ≤ 15ms, and the average inference delay of the edge nodes was 9.3 ms, meeting the requirements for real-time control. In addition, the adjustable range of the virtual inertia constant: 4–12 s; the adjustable range of the damping coefficient: 8–25; the length of the state observation window: 200 ms; the reward weight coefficients α = 0.6 (frequency deviation term), β = 0.4 (steady-state time term); the simulation step size: 50 μs; the training sample size: 1.2 × 10^5^; MAIDDPG critic network node number: 3 × 256, action network hidden layer node number: 2 × 256, learning rate set to 3 × 10^−4^, soft update coefficient τ = 0.005, experience replay buffer capacity is 1 × 10^5^, batch sampling size is 256, discount factor γ = 0.95.

### 2.3. Complete proof of closed-loop stability

The closed-loop system model is composed of the VSG second-order dynamic equation and the MAIDDPG control law. The VSG dynamics can be expressed as shown in Equation (16).


$$\begin{array}{c} J\frac{d\omega }{dt}=P_{m}-P_{e}-D\left(\omega -\omega_{\text{0}}\right) \end{array}$$ (16)

In Equation (16), J represents the virtual inertial constant, ω is the angular frequency (in rad/s), t is the current time, $P_{m}$ is the mechanical power, $P_{e}$ is the electromagnetic power, D is the damping coefficient, and $\omega_{\text{0}}$ is the rated angular frequency. The MAIDDPG control law extracts the current system state through the state observer. The system state vector is shown in Equation (17).


$$\begin{array}{c} s\left(t\right)=\left[\Delta \omega ,\frac{d\Delta \omega }{dt},\Delta P_{m},e_{SOC},P_{wind},P_{\text{pv}}\right] \end{array}$$ (17)

In Equation (17), s(t) represents the system state, Δω is the angular frequency deviation, $\frac{d\Delta \omega }{dt}$ is the rate of change of angular frequency, $\Delta P_{m}$ is the increment of mechanical power disturbance, ${\text{e}}_{\text{SOC}}$ is the error of energy storage charge state, $P_{wind}$ is the wind power, and $P_{pv}$ is the photovoltaic power. The action is output by the actor network to achieve the incremental adjustment of the virtual inertia constant and damping coefficient. This control law aims to minimize the reward function, and the reward function is defined as Equation (18).


$$\begin{array}{c} R\left(t\right)=-\alpha \left|\Delta \omega \left(t\right)\right|-\beta \times t_{settle} \end{array}$$ (18)

In Equation (18), R(t) represents the reward function, α is the penalty weight for angular frequency deviation, and $t_{settle}$ is the time for frequency to return to stability. The entire closed-loop system is driven by the MAIDDPG agent, and by adjusting the VSG parameters in real time, the system can quickly restore frequency stability after disturbances, while also considering the dynamic response speed and steady-state accuracy. The Lyapunov function is shown in Equation (19).


$$\begin{array}{c} V\left(\Delta \omega ,\frac{d\Delta \omega }{dt}\right)=\frac{\text{1}}{\text{2}}J{\left(\Delta \omega \right)}^{\text{2}}+\frac{\text{1}}{\text{2}}{\left(\frac{d\Delta \omega }{dt}\right)}^{\text{2}} \end{array}$$ (19)

In Equation (19), V(·) represents the Lyapunov function. By substituting it into the VSG dynamic equation and the MAIDDPG control law, Equation (20) can be obtained.


$$\begin{array}{c} \begin{array}{c} \frac{\text{d}V}{dt}=-D{\left(d\Delta \omega /dt\right)}^{\text{2}}+\Delta \omega \cdot \Delta P_{e}+\\ \Delta \omega \cdot \Delta J\cdot \Delta \omega /dt+\Delta \omega \cdot \Delta D\cdot \left(\omega -\omega_{\text{0}}\right) \end{array} \end{array}$$ (20)

In Equation (20), $\Delta P_{m}$ and $\Delta P_{e}$ represent the perturbation components of mechanical power and electromagnetic power respectively, while ΔJ and ΔD are the parameter increments output by MAIDDPG. By reasonably designing the control law, ensuring that dV/dt ≤ 0, the system is guaranteed to be stable in the Lyapunov sense. MAIDDPG is guided by the reward function, making the adjustment directions of ΔJ and ΔD always opposite to the system state gradient, thereby suppressing the positive contributions in dV/dt. Thus, the closed-loop system can still asymptotically converge to the equilibrium point under disturbances. The theorem ensuring boundedness and convergence of the system is as follows. Considering the closed-loop system composed of the VSG dynamic equation and the MAIDDPG control law, let the Lyapunov function be as defined previously, and the MAIDDPG control law ensures that dV/dt ≤ -γV + ε, where γ > 0 is the convergence rate and ε > 0 is the upper bound of bounded disturbances. Then, the system state converges to the compact set Ω = {x: V(x) ≤ ε/γ} within a finite time, and the frequency deviation Δω and the rate of angular frequency change dΔω/dt are both bounded. This theorem indicates that even in the presence of model uncertainties and external disturbances, the designed MAIDDPG control law can still ensure that the system state remains bounded and eventually converges to a small neighborhood near the equilibrium point.

During the mapping process of the electromechanical VSG differential equations to the initial actor network weights, the state variables (e.g., Δω, Δδ) and their derivatives in the electromechanical VSG differential equations are treated as input features of the initial actor network. These features are mapped through the neural network to the adjustment values of control parameters ΔJ and ΔD. When initializing the network weights, a random orthogonal matrix is employed to ensure the stability of gradient propagation, while the physical constraints in the VSG differential equations (e.g., damping coefficient range, upper limit of inertia variation rate) are encoded as boundaries of the activation function in the output layer.

## 3. Results and analysis

### 3.1. Verification of VSG adaptive parameter control strategy

Rule-based inertia and damping control strategies were established according to the VSG dynamic response characteristic curves. To verify the effectiveness of this strategy, this study constructed an islanded microgrid simulation model with distributed power sources based on the MATLAB platform. This model incorporated a VSG strategy with typical operating parameters. To ensure the reliability and reproducibility of experimental results, all simulations were executed on the same high-performance computing workstation. In the above simulation environment, this study tested the frequency support capability of VSG under large step changes in power commands, with fixed-parameter VSG as the comparison method. To ensure the scientific validity of experiments, this study used the same computer and selected high-configuration hardware and software equipment to test the algorithm. The data used in the experiment include system frequency, active power, reactive power, voltage amplitude and internal state variables of the VSG. The sampling frequency is 10 kHz, and the simulation duration is 10 seconds. The power step occurs at the 3rd second, with an amplitude of 50% of the rated power. The inertia of the fixed parameter VSG is set as J = 0.1 kg·m², and the damping coefficient D = 20 N·m·s/rad. Under the adaptive strategy, the inertia is dynamically adjusted within the range of 0.5 to 2.0 kg·m², and the damping coefficient adaptsively changes within the range of 10–50 N·m·s/rad. The data set is sourced from the MATLAB/Simulink simulation platform, and is generated by building an isolated island microgrid model containing photovoltaic, energy storage and load. Specific experimental environment configurations are shown in Table 1.

**Table 1:** Specific experimental environment configuration.

/	Configuration item	Detailed information
Hardware	CPU	Intel Xeon E52680 v4, 14 cores, 28 threads, 2.4GHz clock frequency, dynamic acceleration frequency 5.3GHz
	GPU	NVIDIA GeForce RTX 3080, 10GB GDDR6X video memory, core frequency 1440MHz, maximum Rui frequency 1710MHz
	RAM	64GB DDR4, 3200MHz
	Storage	1TB NVMe SSD, 2TB SATA SSD
Software	Operating system	Ubuntu 20.04 LTS, 64-bit
	Programming language	Python 3.8

To test the dynamic response of the strategy during sudden load changes, this study set up two groups of experiments. Power step disturbances were applied at 0.5 s, with one group increasing from the initial value to 12 kW and the other decreasing to 5 kW. The study then tested the changes in system frequency response under different parameter control strategies. Specific experimental results are shown in Fig 8.

**Fig 8:**
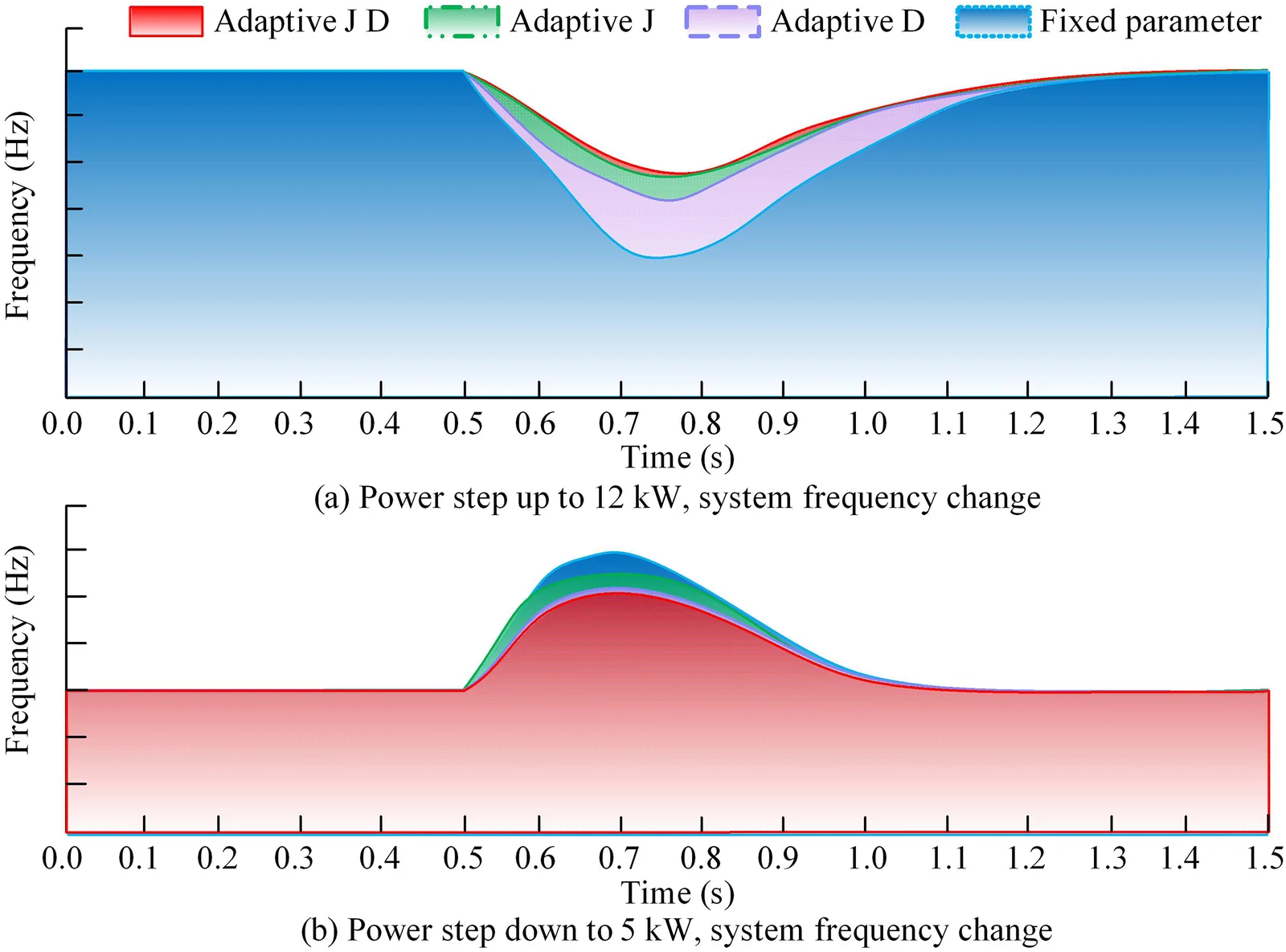
VSG strategy frequency response curves under different strategies.

In Fig 8(a), when the system power command stepped from 5 kW to 12 kW, the system frequency under fixed-parameter VSG control dropped to a minimum of 49.59 Hz, and significant fluctuations occurred during the frequency recovery process. With only adaptive inertia J, the frequency minimum increased to 49.76 Hz, but the curve during recovery was relatively gradual. With only adaptive damping coefficient D, the improvement in frequency minimum was limited. The VSG strategy under adaptive parameter control maintained the frequency minimum at 49.78 Hz, and the curve during frequency recovery was relatively smooth. In Fig 8(b), the frequency peak under the parameter control strategy with adaptive J and D remained below 50.21 Hz, and the recovery process was faster and more stable than that of the comparison strategies. All the above experimental results demonstrated that the adaptive parameter control strategy proposed in this study could significantly improve system stability under sudden disturbances. Subsequently, this study analyzed the VSG active power output curves after applying power command step disturbances at 0.5 s to evaluate its dynamic response performance. Specific experimental results are shown in Fig 9.

**Fig 9:**
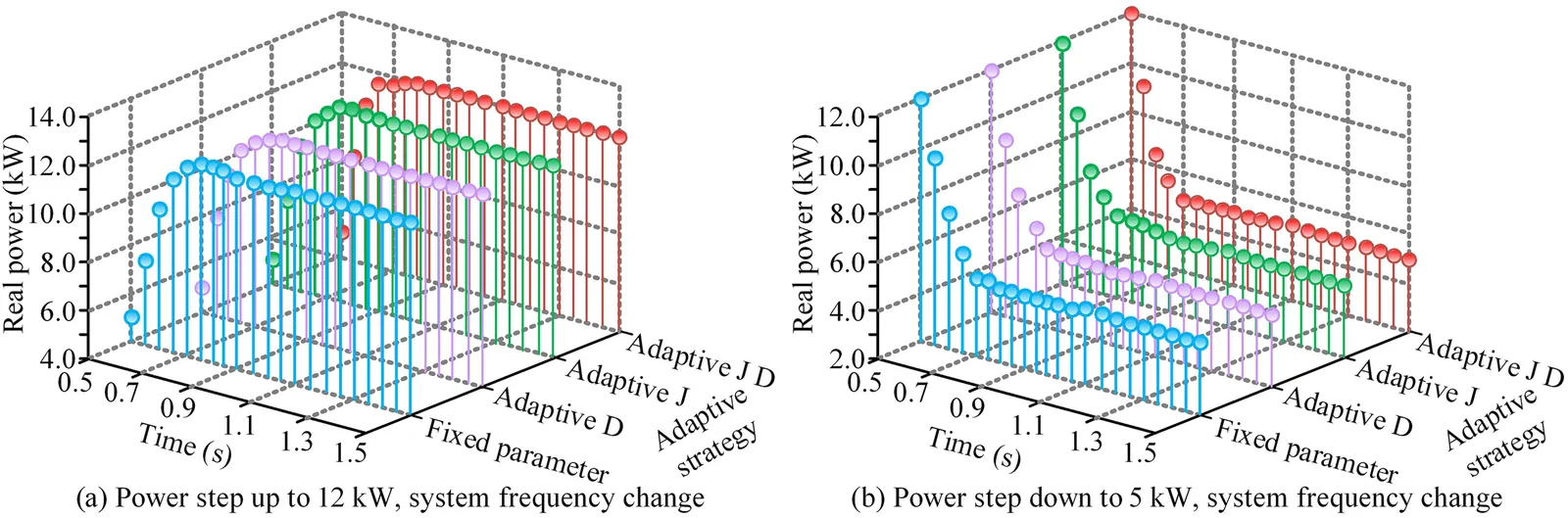
VSG active power output curves under different strategies.

In Fig 9(a), when power stepped from 5 kW to 12 kW, the VSG active power maximum under fixed-parameter control strategy reached 12.10 kW with sustained oscillations. In contrast, simultaneous adaptation of J and D not only limited the peak to 11.98 kW but also recovered to a stable state earlier at 0.9 s. As shown in Fig 9(b), when power suddenly dropped from 12 kW to 5 kW, fixed-parameter VSG experienced a sharp drop to 4.95 kW, while the simultaneous J and D adaptive strategy proposed in this study only dropped to 5.01 kW and recovered to a stable state at 0.9 s. All the above experimental data demonstrated that the strategy of simultaneously adapting J and D could achieve superior control performance. Finally, this study conducted a comparative analysis of the maximum frequency deviation of parameter control strategies under different State of Charge (SOC) conditions. Specific experimental results are shown in Fig 10.

**Fig 10:**
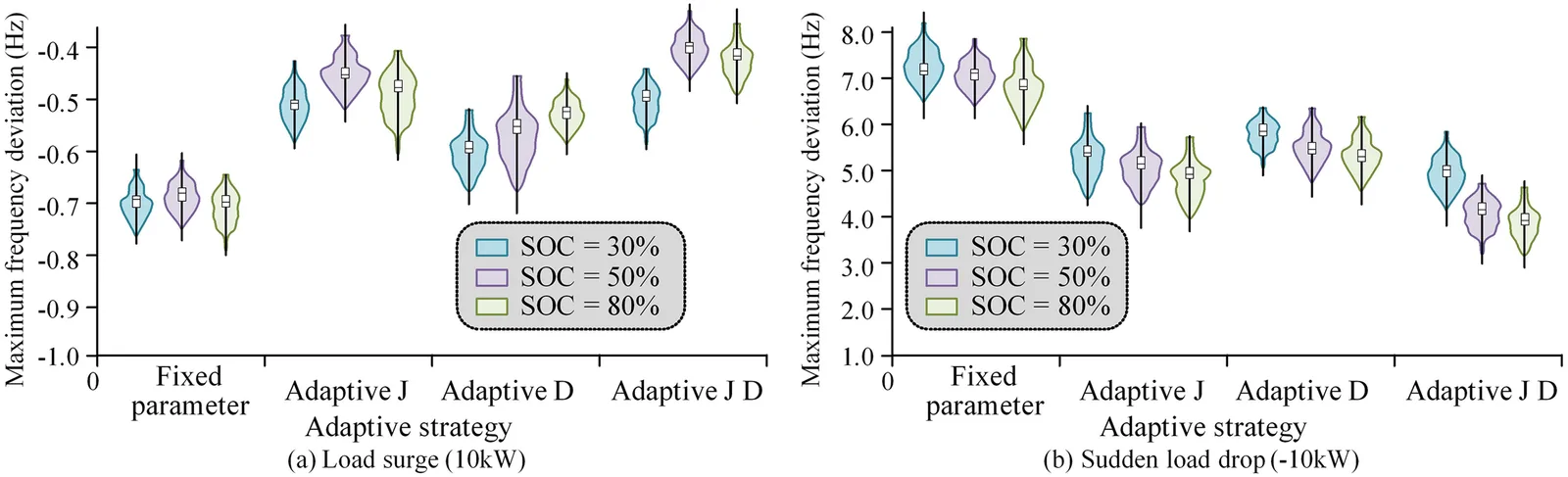
Maximum frequency deviation comparison under different SOC conditions.

As shown in Fig 10(a), when load suddenly increased by 10 kW, the fixed-parameter VSG system had a maximum frequency error of −0.69 Hz at 50% SOC, while the VSG system with simultaneous J and D adaptive strategy had a maximum frequency error of only −0.41 Hz, significantly lower than other control strategies. As shown in Fig 10(b), when system load suddenly decreased by 10 kW, the simultaneous J and D adaptive strategy showed similar performance, with a maximum frequency error of only 0.41 Hz at 80% SOC, while the optimal fixed-parameter VSG system had a maximum frequency error of 0.69 Hz, significantly higher than the adaptive parameter control strategy proposed in this study. All these experimental data indicated that the adaptive parameter adjustment strategy proposed in this study could provide optimal frequency stability under different disturbance scenarios.

### 3.2. Application verification of the VSG-MAIDDPG control strategy

To investigate the changes in voltage peak under three conditions: an increase of 50 kilowatts of load, a sudden reduction of 100 kilowatts of power, and a three-phase short circuit fault, the study recorded the recovery time and voltage peak changes of each method under these three working conditions. The results are shown in Fig 11.

**Fig 11:**
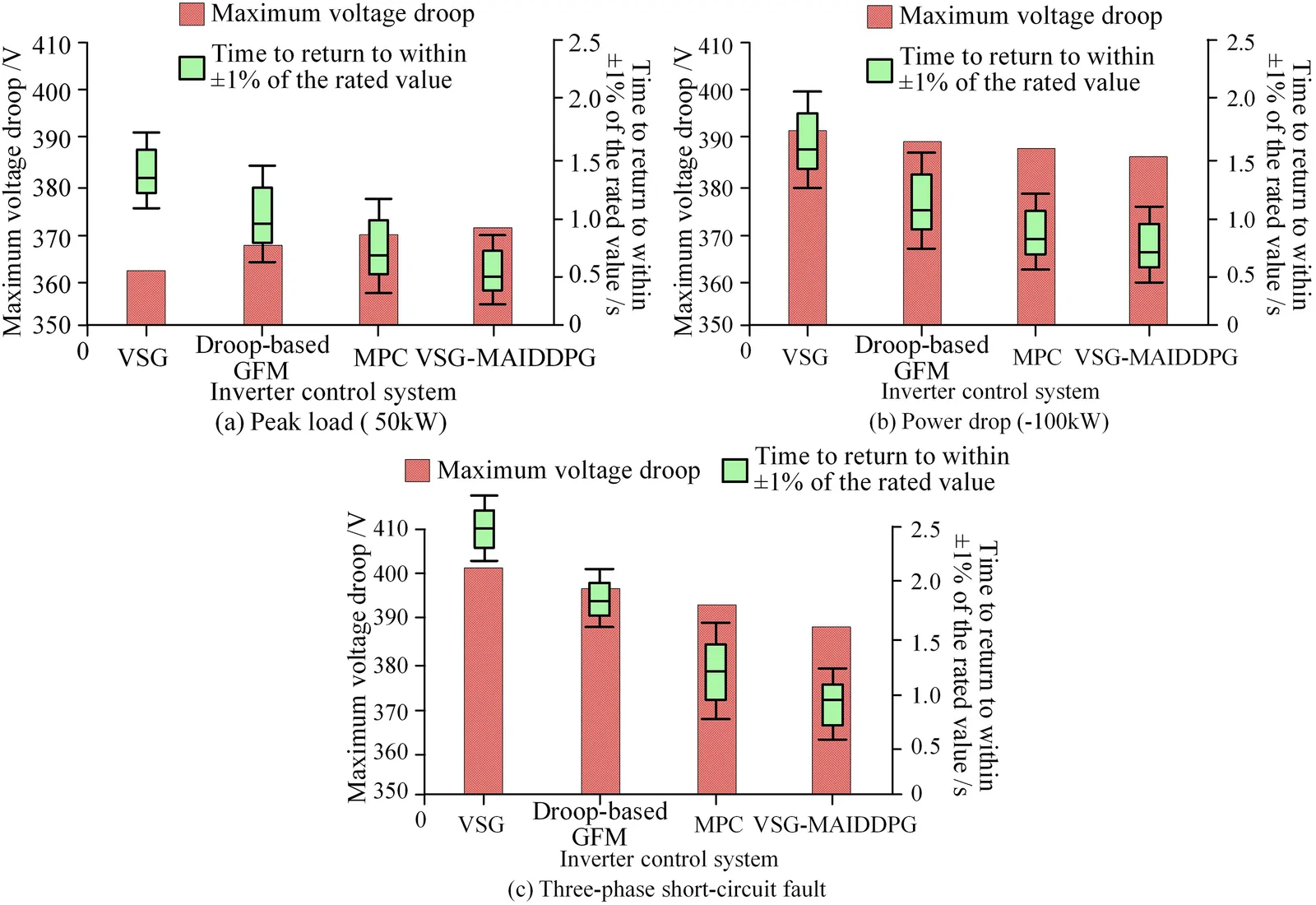
Grid-connected point voltage deviation comparison.

As shown in Fig 11(a), Under 50 kW load increase, traditional VSG control dropped voltage to 362.5 V, Droop-based GFM raised it to 368.9 V, and VSG-MAIDDPG stabilized at 371.7 V with 0.50 s recovery time. For 100 kW power decrease (Fig 11(b)), Droop-based GFM suppressed voltage to 389.5 V, MPC to 388.0 V, and VSG-MAIDDPG to 386.7 V with 0.70 s recovery. Under three-phase short-circuit (Fig 11(c)), VSG-MAIDDPG peaked at 388.5 V and recovered in 0.90 s. VSG-MAIDDPG showed best performance in response and recovery. ITAE cumulative trends under load increase and decrease were analyzed to evaluate strategy smoothness and rapidity. Comparison results of ITAE cumulative processes for four strategies under different power disturbance scenarios are shown in Fig 12.

**Fig 12:**
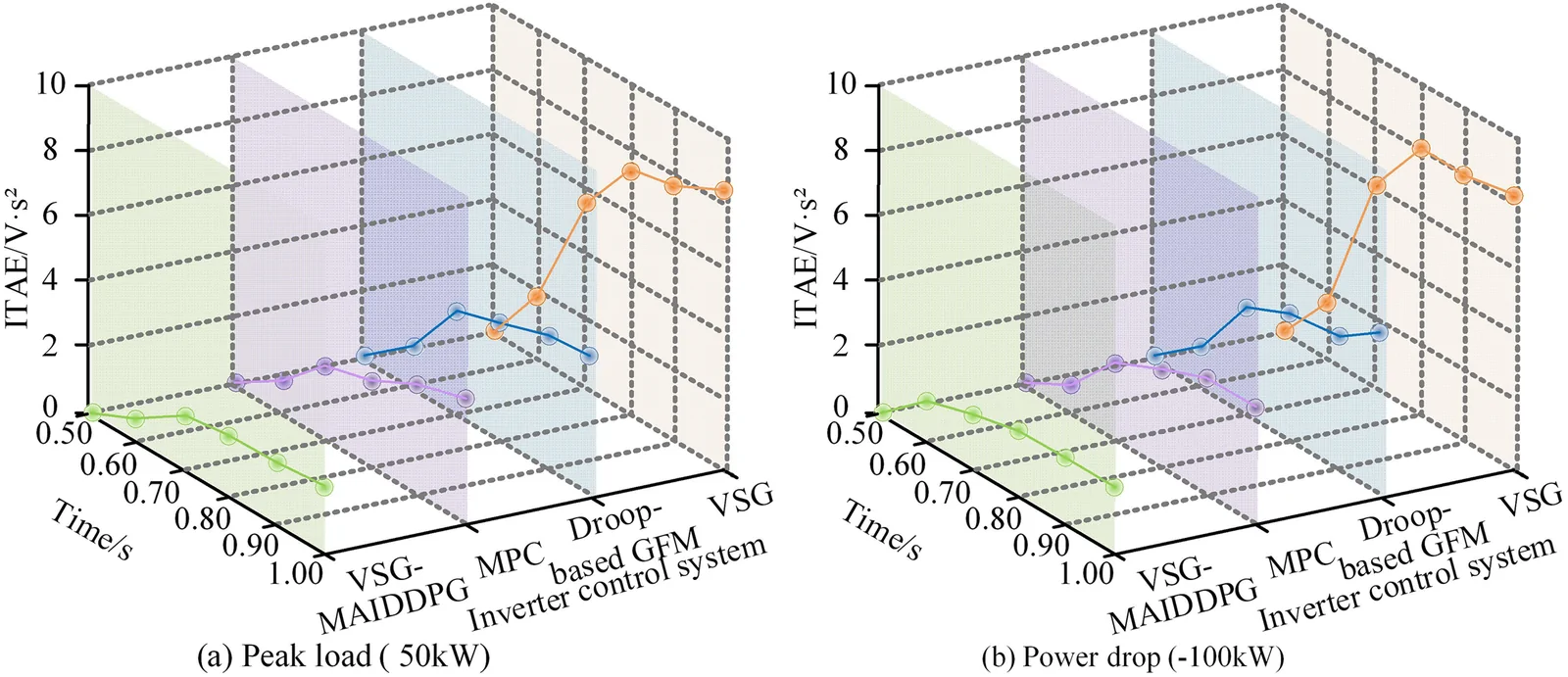
Strategy ITAE cumulative comparison under different disturbance scenarios.

As shown in Fig 12(a), when load suddenly increased by 50 kW, the ITAE value of traditional VSG continued to increase rapidly, reaching 3.98 V·s^2^ at 0.65 s and finally stabilizing at 8.25 V·s^2^. The cumulative process of VSG-MAIDDPG was the most gradual, with a final ITAE value of 1.82 V·s^2^. As shown in Fig 12(b), under the photovoltaic power sudden decrease scenario, Droop-based GFM reached 4.25 V·s^2^, MPC controller reached 3.10 V·s^2^, while VSG-MAIDDPG remained optimal, with ITAE basically converging to 1.85 V·s^2^ at 0.8 s, significantly lower than other control strategies. All the above experimental data demonstrated that VSG-MAIDDPG had the most gradual ITAE rise curve and the best control performance. Subsequently, to evaluate the disturbance rejection performance of control strategies in response to sudden power changes, this study analyzed the dynamic response processes of DC bus voltage and grid-connected point AC voltage under different control strategies during sudden load increase. Specific experimental results are shown in Fig 13.

**Fig 13:**
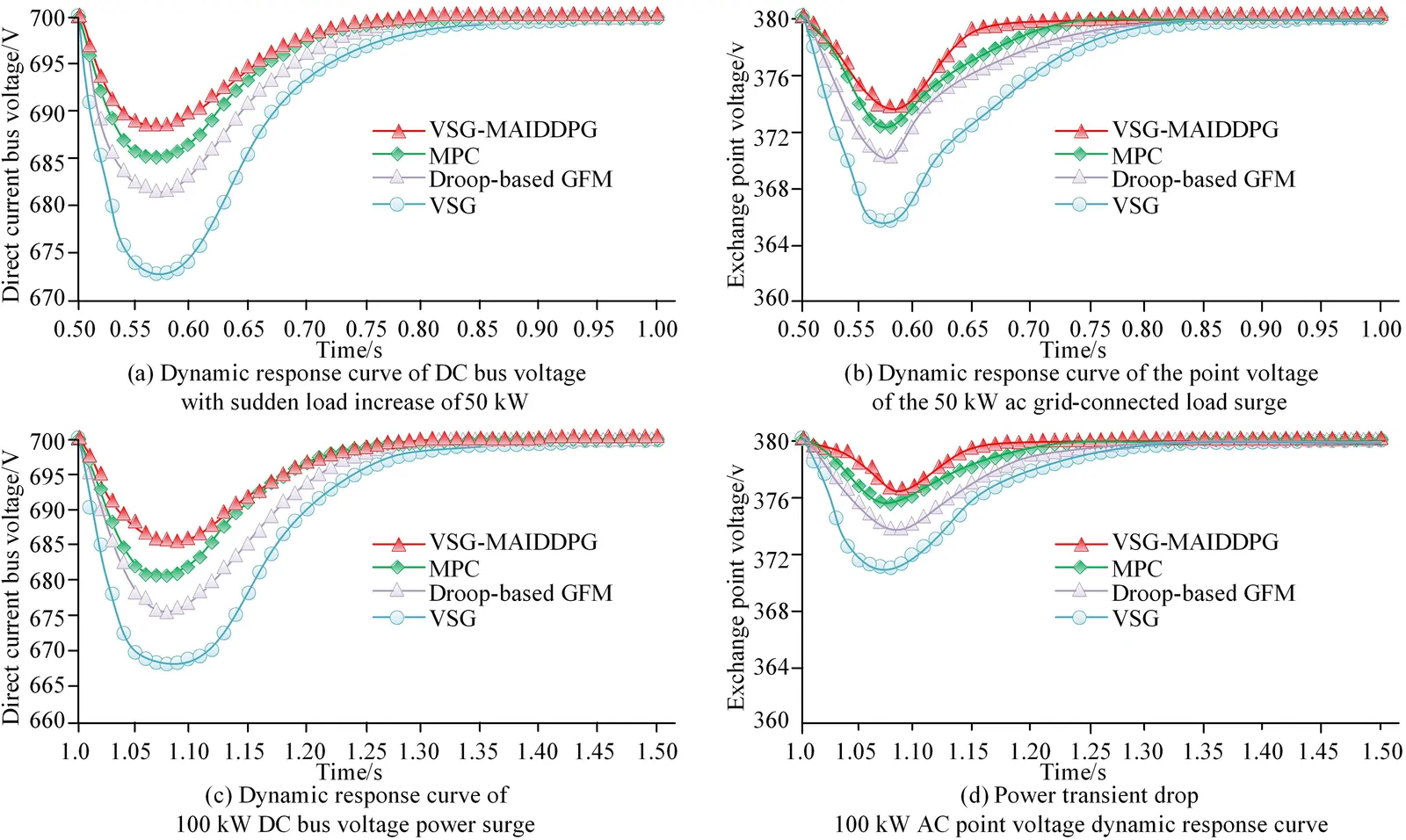
DC bus voltage and AC voltage change comparison.

In Fig 13(a), during a sudden load increase, DC bus voltage with traditional VSG dropped to 672.5 V at 0.58 s, while VSG-MAIDDPG only fell to 687.5 V. Fig 13(b) shows AC voltage data: traditional VSG had a more pronounced drop, whereas VSG-MAIDDPG maintained a minimum of 374.5 V and recovered to 379.8 V by 0.80 s, closest to rated voltage. When power suddenly decreased by 100 kW, VSG-MAIDDPG performed best, dropping only to 685.5 V (Fig 13(c)); it recovered to 696.5 V by 1.20 s, showing the fastest recovery. For AC grid-connected point voltage (Fig 13(c) and Fig 13(b)), VSG-MAIDDPG's minimum was 377.2 V, recovering to 380.2 V by 1.30 s, nearest to rated voltage. These results demonstrate VSG-MAIDDPG's superior control performance. The study also analyzed dynamic response time, steady-state accuracy, oscillation suppression, and strategy efficiency across control strategies, using metrics such as ±2% tolerance for response time and ITAE for efficiency in suppressing cumulative frequency deviation. Specific experimental results are shown in Table 2.

**Table 2:** Comparison of dynamic response time, steady-state accuracy, oscillation suppression capability, and efficiency for different strategies.

Indicator		Inverter control strategy			
		VSG	Droop-based GFM	MPC	VSG-MAIDDPG
Dynamic response time (s)	Voltage recovery time	1.85 ± 0.14	1.41 ± 0.16	0.82 ± 0.05	0.61 ± 0.05^*#^^
	Power stability time	2.14 ± 0.19	1.47 ± 0.10	0.96 ± 0.09	0.71 ± 0.08^*#^^
Steady-state accuracy (%)	Voltage stability precision	98.21 ± 0.25	98.95 ± 0.22	99.24 ± 0.20	99.63 ± 0.19^*#^^
	Frequency stability accuracy	99.14 ± 0.16	99.48 ± 0.17	99.72 ± 0.11	99.84 ± 0.09^*#^^
Oscillation suppression capability	Overshoot (%)	6.41 ± 0.07	3.67 ± 0.06	3.05 ± 0.05	2.11 ± 0.04^*#^^
	Number of adjustments	4.25 ± 0.10	2.63 ± 0.12	2.14 ± 0.10	1.73 ± 0.13^*#^^
System efficiency (%)		95.84 ± 0.10	Droop-based GFM	97.25 ± 0.09	97.83 ± 0.09^*^^

Note: In Table 2, ^*^ indicates that there is a significant difference between the VSG-MAIDDPG and VSG results, with *p* < 0.05; # indicates that there is a significant difference between the VSG-MAIDDPG and Droop-based GFM results, with *p* < 0.05; ^ indicates that there is a significant difference between the VSG-MAIDDPG and MPC results, with *p* < 0.05.

In dynamic response, VSG-MAIDDPG achieved the best voltage recovery times of 0.61 s and 0.71 s, outperforming traditional VSG (1.85 s), Droop-based GFM (1.41 s), and MPC controller (0.82 s). It also showed the highest steady-state accuracy in voltage (99.63%) and frequency (99.84%), the lowest overshoot in oscillation suppression (2.11%), and the highest strategy efficiency (97.83%), demonstrating clear advantages. A comprehensive evaluation then assessed reliability and generalization under near-practical conditions, with robustness against weak grid adaptability, parameter uncertainty, measurement noise, and non-ideal communication compared statistically in Table 3.

**Table 3:** Verification Results of Robustness and Non-Ideal Operating Conditions for Different Control Strategies.

Test Category	Specific Condition	Evaluation Metric	Comparative Results of Control Strategies			
			VSG	Droop-based GFM	MPC	VSG-MAIDDPG
Weak‑Grid Adaptability	Short‑Circuit Ratio SCR = 3.0	Damping ratio of dominant mode	0.10	0.15	0.21	0.25
		Steady‑state voltage deviation (%)	2.85	2.10	1.60	0.98
	Short‑Circuit Ratio SCR = 2.0	Damping ratio of dominant mode	0.06	0.0.9	0.13	0.17
		System stability	Unstable	Marginally stable	Stable	Stable
Parameter Uncertainty	Line inductance +20%	Increase in frequency recovery time (%)	+31.5	+19.5	+14.2	+7.5
	VSG rated capacity –15%	Increase in power overshoot (%)	+25.4	+16.1	+11.3	+5.8
Measurement‑Noise Immunity	Frequency measurement with noise (SNR = 35dB)	Control‑output oscillation amplitude (pu)	0.075	0.058	0.042	0.022
	Power measurement with noise	Variance of parameter‑adjustment command (10^−3^)	4.21	2.87	1.95	0.89
Non‑ideal Communication	Information‑synchronization delay 50ms	Performance Degradation(ITAE, %)	Failed	+38.2	+16.5	+8.1
	Packet‑loss rate 5%	Extension of system recovery time (%)	–	+52.1	+24.3	+11.6

As shown in Table 3, under weak grid conditions with a short-circuit ratio reduced to 2.0, the traditional VSG becomes unstable. The Droop-based GFM with a damping ratio of 0.09 reaches a critical state, while the MPC strategy maintains stability at a damping ratio of 0.13. The VSG-MAIDDPG strategy achieves a damping ratio of 0.17, ensuring system stability and optimal dynamics. When line reactance increases by 20%, the frequency recovery time increases by 31.5%, 19.8%, 14.2%, and 7.5% for each strategy, respectively. With 35 dB measurement noise introduced, the VSG-MAIDDPG control output jitter was only 0.022 pu, significantly lower than the traditional VSG. Under 50 ms communication delay, the ITAE performance degradation of VSG-MAIDDPG was 8.1%, far below the 38.2% degradation of the adaptive VSG. This demonstrates that the VSG-MAIDDPG strategy exhibits the strongest robustness and performance retention across various non-ideal operating conditions. Finally, to validate the applicability of the proposed VSG-MAIDDPG multi-agent control strategy in practical large-scale heterogeneous systems, performance tests were conducted across different VSG scales. The experimental results are shown in Table 4.

**Table 4:** Statistical Summary of VSG-MAIDDPG Policy Scalability Verification Results.

Number of VSGs in Parallel	Evaluation Metric	VSG-MAIDDPG
3	ITAE (Hz·s²)	1.92
	Training Steps to Convergence (×10^3^)	48.50
	Average Single-Step Decision Time (ms)	3.20
6	ITAE (Hz·s²)	2.15
	Training Steps to Convergence (×10^3^)	105.30
	Average Single-Step Decision Time (ms)	6.80
9	ITAE (Hz·s²)	2.41
	Training Steps to Convergence (×10^3^)	187.60
	Average Single-Step Decision Time (ms)	10.5
12	ITAE (Hz·s²)	2.73
	Training Steps to Convergence (×10^3^)	285.40
	Average Single-Step Decision Time (ms)	14.3

As shown in Table 4, when the system scale expands from 3 VSGs to 15 VSGs, the ITAE metric characterizing frequency regulation accuracy increases gradually from 1.92 Hz·s² to 3.05 Hz·s², indicating that the algorithm maintains effective coordination capabilities even in ultra-large-scale parallel scenarios. Concurrently, the algorithm's training cost exhibits sublinear growth: the number of steps required for training convergence increases from 48.5 × 10^3^ steps to 398.2 × 10^3^ steps, while the time per decision step rises from 3.20 ms to 18.10 ms. These findings demonstrate that the VSG-MAIDDPG strategy employed in this study effectively controls the growth rate of computational complexity with increasing VSG scale, indicating its engineering applicability.

To verify the stability and practical application effect of the control strategy proposed in the research (Strategy 1), experiments were conducted to compare it with the control strategies in References [29] (Strategy 2) [30], (Strategy 3), and [31] (Strategy 4). The four strategies were respectively deployed on the same microgrid experimental platform. Comparative experiments were carried out under three typical conditions: a sudden increase of 50% load, a sudden removal of 70% load, and a three-phase short-circuit fault. The comparison indicators included frequency deviation, the number of protection locking, voltage drop depth, recovery time, and the number of abnormal warnings. The specific results are shown in Table 5.

**Table 5:** Comparative experimental results of different literature control strategies.

Project		Frequency deviation (Hz)	Number of protection lockouts (times)	Depth of voltage sag (%)	Recovery time (h)	Number of abnormal warnings (times)
Increase load by 50%	Strategy 1	0.12	0	4.3	0.61	0
	Strategy 2	0.28	2	9.7	1.24	5
	Strategy 3	0.21	1	7.2	0.93	3
	Strategy 4	0.35	3	11.6	1.57	7
Remove 70% of the load	Strategy 1	0.15	0	5.1	0.68	1
	Strategy 2	0.33	2	10.4	1.32	4
	Strategy 3	0.26	1	8.5	1.02	2
	Strategy 4	0.39	3	12.3	1.65	6
Three-phase short circuit fault	Strategy 1	0.32	1	18.6	2.35	4
	Strategy 2	0.57	2	23.1	2.89	12
	Strategy 3	0.49	1	20.4	2.62	9
	Strategy 4	0.63	3	25.8	3.17	13

As shown in Table 5, Control Strategy 1 demonstrates the best overall performance in all three typical operating conditions. In scenarios of load sudden increase and sudden removal, the frequency deviation is the smallest, the protection locking is zero or the lowest, the average voltage drop depth is reduced by approximately 36%, and the recovery time is shortened by nearly 40%; even in the severe three-phase short-circuit fault, the number of abnormal warnings is 55.6% less than that of the suboptimal strategy (Strategy 3), and no cascading protection action is triggered. This indicates that the proposed strategy can achieve dual dynamic decoupling control of the system's frequency and voltage, thereby initiating a multi-time-scale collaborative response mechanism immediately upon the occurrence of disturbances.

The proposed model in the study combines the advantages of the physical model and reinforcement learning, achieving complementarity in dynamic decoupling control and multi-time-scale coordinated response. To verify the individual contributions of these mechanisms, the study designed ablation experiments. The comparison ablation groups included removing the physical model initialization (only retaining the reinforcement learning framework) (ablation 1), removing the dynamic decoupling module (only retaining a single frequency control) (ablation 2), removing the multi-time-scale coordinated mechanism (only retaining a single time-scale response) (ablation 3), standard random initialization (ablation 4), and the complete model (complete group). The comparison indicators included convergence speed, final reward, voltage recovery time, and the number of abnormal warnings. The results are shown in Table 6.

**Table 6:** Results of the ablation experiment.

Control Group	Convergence Speed (Rounds)	Final Reward	Voltage Recovery Time (ms)	Number of Abnormal Warnings
Complete group	32	0.91	12.4	2
Ablation Group 1	55	0.74	16.2	5
Ablation Group 2	48	0.69	18.7	8
Ablation Group 3	51	0.72	15.8	6
Ablation Group 4	68	0.63	20.5	11

As shown in Table 6, the complete model significantly outperforms all the ablation groups in all indicators. Compared with Ablation 1, the convergence speed of the complete model has increased by approximately 41.8%, the final reward is 23.0% higher, the voltage recovery time is shortened by 23.5%, and the number of abnormal warnings is reduced by 60.0%. This indicates that the physical model initialization not only accelerates the learning process but also significantly enhances the robustness and safety of the control strategy. Compared with Ablation 2, the complete model shortens the voltage recovery time by 33.7%, reduces the number of abnormal warnings by 75.0%, verifying the crucial role of the dynamic decoupling module in accurately separating different frequency disturbances and avoiding interference between control actions. Compared with Ablation 3, the complete model has a convergence speed increase of 37.3%, a final reward 26.4% higher, a voltage recovery time shortened by 21.5%, and the number of abnormal warnings reduced by 66.7%, demonstrating that the multi-time-scale collaborative mechanism can effectively coordinate control actions with different response speeds and avoid system oscillation or over-tuning caused by response mismatch.

## 4. Discussion

With the continuous promotion of renewable energy, the problem of decreasing strategy inertia caused by the grid connection of distributed power sources such as photovoltaic and wind power through inverters has become increasingly prominent. The proposed method stabilizes the frequency nadir and peak frequency at superior levels in the scenario of sudden load increase, effectively delaying the trend of frequency collapse; in the case of sudden load shedding, its probability error is also significantly lower than that of the comparison method. The superiority of this strategy lies in the fact that the real-time adjustment of adaptive inertia effectively enhances the inertia support capacity of the strategy. On the other hand, the adaptive damping coefficient suppresses power oscillations, enabling the strategy to converge more quickly. The synergy of the two effects ensures the frequency stability during the transient process of VSG and improves the accuracy of power control. In existing research, Lu S's team proposed a frequency-domain analysis method for VSG parameter coupling, revealed the mechanism of key parameter coupling on low-frequency oscillations, and verified the effectiveness of the theoretical model through simulation experiments [32]. However, their research focused on mechanism analysis and failed to propose specific parameter solutions. Lei J et al. quantitatively evaluated the impact of damping on VSG control performance in their study, adopted the damping area approximation method to quantitatively assess the attraction domain, and finally verified the effectiveness of this method through experiments [33]. However, this method still failed to fully consider the dynamic adaptability of inertia parameters. In contrast, this study developed a complete coordinated adaptive control strategy for inertia and damping, simultaneously addressing two key problems: inertia support and oscillation suppression.

In addition, the research addresses the issue that it is difficult to achieve global optimal control in the parameter control strategy for distributed power generation grid connection. By adopting the improved MAIDDPG algorithm, multiple VSG intelligent agents can output continuous control quantities in complex and dynamic environments, and a wind-solar-storage power generation inverter regulation strategy based on VSG-MAIDDPG is constructed. The outstanding performance of this strategy is attributed, on the one hand, to the collaborative adaptive adjustment of inertia and damping coefficients by the intelligent decision-making strategy of VSG-MAIDDPG, and on the other hand, to the ability of MAIDDPG algorithm to form advanced control capabilities for complex conditions through learning from historical data. Tan L et al. optimized virtual inertia and damping initial values through an improved particle swarm algorithm and introduced an inertia component in the control. Simulation experiments showed that this strategy could significantly reduce frequency overshoot and oscillations, but lacked online adjustment capability [34]. Ling Z et al. proposed using multi-radial basis function neural network to adaptively adjust the virtual inertia and damping of VSG. Experimental results showed that the control strategy incorporating multi-radial basis function neural network could effectively suppress oscillations, but the network structure and training mechanism remained relatively complex [35]. In contrast, the introduction of the MAIDDPG algorithm resolves the challenge of globally optimizing parameters for multi-VSG strategies in dynamic environments. It overcomes the limitations of local optimization by individual agents and demonstrates significant advantages in handling complex disturbances and large-scale systems through validated robustness and scalability.

## 5. Summary

To address the issue of insufficient voltage regulation capability of traditional inverters under complex operating conditions, a wind-solar-storage power generation inverter control strategy based on VSG-MAIDDPG was proposed. The improved MAIDDPG algorithm was used to achieve coordinated parameter optimization for multiple VSG units in complex operating environments. Simulation experiments showed that this method could control the minimum frequency to 49.78 Hz and limit the maximum frequency to 50.21 Hz in the scenario of sudden load increase. Under the 50% SOC condition, the maximum frequency error of this method was only −0.41 Hz, maintaining the optimal performance. In the case of sudden load increase, this strategy stabilized the voltage deviation at 371.7V and shortened the recovery time to 0.50s. In power disturbance scenarios, this strategy limited the voltage fluctuations to 386.7 V and still maintained the fastest recovery speed. The ITAE results showed that VSG-MAIDDPG achieved the optimal values of 1.82V/s^2^ and 1.85V/s^2^ in the scenarios of sudden load increase and sudden drop in photovoltaic power. Meanwhile, the steady-state accuracy of VSG-MAIDDPG reached 99.63%, and the strategy efficiency reached 97.83%.

Based on the results of the above experiments, it can be concluded that the proposed VSG-MAIDDPG strategy outperforms the comparison methods in key indicators such as frequency, voltage, and recovery time, fully verifying its effectiveness and robustness in complex conditions. However, there are still limitations at present. In the future, it is considered to further explore the adaptability of the algorithm in extreme weather scenarios and introduce a lightweight network structure to reduce computational complexity, in order to improve its deployment efficiency on edge computing devices. At the same time, a virtual simulation environment can be constructed by combining digital twin technology to enhance the offline training efficiency and online migration capability of the strategy, thereby accelerating the process of transforming the algorithm from theoretical verification to practical engineering application.
